# Representation of women on National Institutes of Health study sections before and during COVID-19 pandemic

**DOI:** 10.1017/cts.2025.10091

**Published:** 2025-07-07

**Authors:** Lucy O. Alejandro, Alexandra Knitter, Monica Kowalczyk, Wen Wan, Valerie G. Press, Vineet M. Arora, Anna Volerman

**Affiliations:** 1 Department of Medicine, University of Chicago, Chicago, IL, USA; 2 Department of Pediatrics, University of Chicago, Chicago, IL, USA

**Keywords:** Grant review, representation, research, workforce, women

## Abstract

Women remain underrepresented in National Institutes of Health (NIH) study sections, panels of scientists who review grant applications to inform national research priorities and funding allocations. This longitudinal, retrospective study examined the representation of women on study sections before and during the COVID-19 pandemic. Overall, 16,902 reviewers served on 1,045 study sections across 2019, 2020, and 2021, of which 40.1% (*n* = 6,786) were women. The likelihood of reviewers being women significantly increased from 2019 to 2021, except among chairpersons. Understanding the representation of scientists influencing NIH grant decisions is important to ensuring scientific discovery that meets the nation’s pluralistic needs.

## Introduction

Although women represent half of medical students and biomedical doctoral graduates, they remain underrepresented in influential roles in biomedicine, including faculty and leadership positions [1]. An important but overlooked metric of representation is within the National Institutes of Health (NIH) study sections. These panels of scientists conduct peer review and score grant applications, informing national research priorities and funding allocations. A novel study found that women were underrepresented in NIH study sections in 2019 and were less likely to be on study sections that resulted in greater funding and research grants awarded, suggesting less influential opportunities to impact the nation’s research agenda [2].

The representation of women in NIH study sections is important because women bring unique perspectives that enhance the breadth and depth of biomedical discovery [1]. Past research suggests that more diverse teams promote creativity, innovation, and problem solving while minimizing the effects of groupthink and bias [3]. The representation of women in scientific research is also inextricably linked to innovations in women’s health. For instance, research shows that women scientists are more likely than male scientists to report and assess sex and gender differences [4,5]. They are also more likely to spearhead research and programs focused on female-focused issues or populations, suggesting that who conducts research affects the course of discovery [6,7]. Further, the representation of women among decision-making bodies like NIH study sections may alleviate the chronic underfunding of women’s health research, as evidenced by gaps in funding for conditions that exclusively or disproportionately affect women [8]. Finally, improving the representation of women on study sections may improve the gender gap in NIH funding. While studies find that men and women have near-equal NIH funding success rates, women are less likely to be represented among grant applicants and more likely to leave NIH-funded career pipelines [9]. Serving as a reviewer offers privileges and benefits such as deep knowledge of the peer review process, networking, and grant application extensions. As such, promoting the representation of women in NIH study sections may help reduce attrition and narrow the gender gap in NIH funding.

During the COVID-19 pandemic, women scientists, especially those with caregiving responsibilities, experienced unique challenges and opportunities [10,11]. For example, women were more likely to report decreased productivity and negative impacts on manuscript publications, contributing to slowed career advancement and potential attrition from the academic pipeline [10,11]. Concurrently, women were more likely to report increased flexibility in professional work, suggesting that remote or hybrid opportunities can be beneficial [11]. To our knowledge, no study has investigated longitudinal trends regarding women’s representation in study sections, including before and during the pandemic. This research helps inform future interventions to promote equitable decision-making regarding the future of scientific discovery for our diverse nation.

## Materials and methods

This longitudinal, retrospective study examined chartered and special emphasis study sections from all NIH institutes, centers, and offices during 2019, 2020, and 2021 May to July review cycles. University of Chicago’s Institutional Review Board deemed this study exempt because it used publicly available data. We followed STROBE reporting guidelines.

Data were extracted from NIH study section rosters about each reviewer’s status (permanent, temporary), role (chairperson, member, mail reviewer), and academic rank (professor, associate professor, assistant professor, none or other). Reviewers’ gender (man, woman) was identified using pronouns and/or photos from reputable websites. If unconfirmed (*n* = 49), gender was imputed using Genderize.io (Demografix ApS), a validated algorithm that has been used in past studies to assess gender representation in medicine and science [2,7,12–14]. We used the standard threshold of 60% probability, which was implemented in alignment with past studies [15]. While Genderize.io can be limited by non-classifications [16], this particular study only encountered one individual (*n* = 1) whose gender could not be imputed.

Descriptive statistics were used to summarize the representation of unique women reviewers across years, stratified by status, role, and academic rank. To model the probability of reviewers being women and assess the impact of the COVID-19 pandemic, we used a multilevel model via generalized estimating equation with a log link function and Poisson distribution. The primary analysis compared 2021 to the pre-pandemic year, 2019. The secondary analysis compared 2020 to 2019. Study sections nested within institutes/centers/offices were considered as random clusters and cluster-robust standard errors were estimated. The model was adjusted for each reviewer’s academic rank, status, and role. Adjusted risk ratios (aRR) with 95% confidence intervals (CI) were reported. In addition, we conducted multiple subgroup analyses to check the consistency of the main findings and detect potential heterogeneity. Sensitivity analyses were also conducted excluding individuals whose gender was imputed (*n* = 48). Analyses were conducted using SAS software 9.4 (SAS Institute Inc., Cary, North Carolina).

## Results

Overall, 16,902 unique reviewers served on 1,045 study sections across 2019, 2020, and 2021, of which 40.1% (*n* = 6,786) were women (Table 1). Women represented 34.8% (*n* = 2,926/8,417) of professors, 45.4% (*n* = 2,343/5,150) of associate professors, and 48.3% (*n* = 1,212/2,509) of assistant professors, and 48.3% (*n* = 152/315) of reviewers with no or other academic ranks.

**Table 1.** Representation of women in National Institutes of Health study sections by status, role, and academic rank, 2019–2021 May–June review cycles

	Representation of women, % (no. women/no. total)			
Characteristics	Overall, 2019–2021^a^	2019	2020	2021
All reviewers	40.1 (6,786/16,902)	39.1 (3,246/8,297)	38.9 (2,948/7,576)	41.9 (3,710/8,857)
**Status**				
Permanent	39.9 (4,330/10,848)	38.9 (2,189/5,621)	39.3 (2,078/5,284)	41.5 (2,344/5,648)
Temporary	40.5 (3,049/7,526)	39.4 (1,100/2,792	38.1 (917/2,407)	42.6 (1,404/3,296)
**Role**				
Chairperson	37.4 (327/874)	35.4 (133/376)	33.1 (125/378)	42.0 (160/381)
Member	40.3 (6,562/16,267)	39.3 (3,081/7,839	39.2 (2,812/7,179)	42.0 (3,533/8,414)
Mail reviewer	35.9 (102/284)	34.8 (49/141)	42.6 (23/54)	33.3 (30/90)
**Academic rank**				
Professor	34.8 (2,926/8,417)	33.7 (1,472/4,371)	34.2 (1,357/3,972)	36.2 (1,564/4,320)
Associate professor	45.4 (2,343/5,150)	45.4 (1,070/2,356)	45.7 (933/2,041)	46.9 (1,246/2,654)
Assistant professor	48.3 (1,212/2,509)	48.4 (390/806)	44.6 (402/901)	51.3 (603/1,176)
None/other	48.3 (152/315)	49.2 (61/124)	43.3 (42/97)	50.0 (53/106)

a Total number of overall reviewers across years reflects unique reviewers because some reviewers participated in the same or different study sections in multiple years and may have held different statuses, roles, or academic ranks.

Women represented 39.1% (*n* = 3,246/8,297) of reviewers in 2019, 38.9% (*n* = 2,948/7,576) in 2020, and 41.9% (*n* = 3,710/8,857) in 2021. The likelihood of reviewers being women significantly increased from 2019 to 2021 (aRR = 1.06; CI [1.03–1.10]) (Table 2).

**Table 2.** Likelihood of reviewers on National Institutes of Health study section being women, 2019–2021 May–June review cycles

Subgroup and time period^a^	Risk ratio	95% confidence interval	p-value
**All reviewers (*n* = 26,057)**			
2019	1 [ref]	NC	NC
2020	1.002	0.97, 1.04	0.9
2021	1.06	1.03, 1.10	<0.001
**Permanent (*n* = 17,473)**			
2019	1 [ref]	NC	NC
2020	1.01	0.97, 1.05	0.7
2021	1.06	1.02, 1.10	0.007
**Temporary (*n* = 8,584)**			
2019	1 [ref]	NC	NC
2020	0.99	0.93, 1.05	0.6
2021	1.07	1.01, 1.14	0.02
**Chairperson (*n* = 1172)**			
2019	1 [ref]	NC	NC
2020	0.95	0.80, 1.13	0.6
2021	1.18	0.98, 1.41	0.08
**Non-chairperson (*n* = 24,885)**			
2019	1 [ref]	NC	NC
2020	1.004	0.97, 1.04	0.8
2021	1.06	1.02, 1.09	0.002

Ref = reference group; NC = not calculated.

a Poisson regressions assessed the likelihood that any given reviewer is a woman, adjusting for academic rank, status, and role. Random effects included the study section and the institute/center/office. Fixed effects included year, academic rank, status, and role. Changes over time were also stratified by status (permanent/temporary) or role (chairperson/non-chairperson) where noted. Total *n* for all reviewers and by stratified subgroups reflects all non-unique reviewers.

Women represented 39.9% (*n* = 4,330/10,848) of permanent reviewers and 40.5% (*n* = 3,049/7,526) of temporary reviewers. Among permanent reviewers, women represented 38.9% (*n* = 2,189/5,621) in 2019, 39.3% (*n* = 2,078/5,284) in 2020, and 41.5% (*n* = 2,344/5,648) in 2021. The likelihood of permanent reviewers being women increased significantly from 2019 to 2021 (aRR = 1.06; CI [1.02–1.10]). Among temporary reviewers, women represented 39.4% (*n* = 1,100/2,792) in 2019, 38.1% (*n* = 917/2,407) in 2020, and 42.6% (*n* = 1,404/3,296) in 2021. The likelihood of temporary reviewers being women increased significantly from 2019 to 2021 (aRR = 1.07; CI [1.01–1.14]).

Women represented 37.4% (*n* = 327/874) of chairpersons and 40.3% (*n* = 6,634/16,462) of non-chairpersons (members, mail reviewers). Among chairpersons, women represented 35.4% (*n* = 133/376) in 2019, 33.1% (*n* = 125/378) in 2020, and 42.0% (*n* = 160/381) in 2021. The likelihood of chairpersons being women did not significantly change between 2019 and 2021 (aRR = 1.18; CI [0.98–1.41]). Among non-chairpersons, women represented 39.2% (*n* = 3,127/7,969) in 2019, 39.2% (*n* = 2,834/7,225) in 2020, and 41.9% (*n* = 3,560/8,497) in 2021. The likelihood of non-chairpersons being women significantly increased from 2019 to 2021 (aRR = 1.06; CI [1.02–1.09]).

For the secondary analysis examining changes from 2019 to 2020, there were no significant differences in the likelihood of permanent reviewers, temporary reviewers, chairpersons, or non-chairpersons being women (Table 2). Findings in the sensitivity analyses, where individuals were excluded if their gender was imputed, did not differ for both the primary and secondary analyses (Supplementary Table).

## Discussion

This study is the first to longitudinally examine representation on NIH study sections, which influences funding decisions and biomedical science discovery in our nation. Though women were less than half of all NIH study section reviewers, their representation increased overall between 2019 and 2021. While this increase is statistically significant, it is important to note that the absolute difference is small. In contrast, among chairpersons, changes in women’s representation were not statistically significant across years. However, the absolute difference in the proportion of women chairpersons between 2019 and 2021 was relatively larger than among all other subgroups.

Overall improvements in women’s representation could reflect past NIH efforts to advance diversity, equity, and inclusion in study sections, including building tools to recruit and retain diverse scientists in study sections. One example is the Early Career Reviewer Program, which aims to recruit qualified early career scientists to enrich the pool of study section reviewers; data from the first 12 years of the program show that it has achieved gender parity [17]. Additionally, in 2020, the NIH Center for Scientific Review, which conducts the majority of NIH study sections, hired consultants to assess organizational processes and identify actions to foster transparency and inclusion [18]. Such efforts are commendable and must be continued and expanded, as grant funding allocations should be made by decision-making bodies that represent the diverse populations whom scientific research is meant to serve. Additionally, virtual study sections implemented during the pandemic may have enhanced inclusion of women, who traditionally face greater barriers to travel for in-person meetings like caregiving responsibilities [19]. A prior evaluation conducted by the Center for Scientific Review found that a smaller proportion of women preferred in-person meetings than men [17]. Finally, our findings may be because more women had time to participate in study sections as other professional opportunities, like conference travel, declined and flexibility in work efforts increased [10]. The nonsignificant findings on the representation of women among chairpersons may reflect the longer timelines needed for progress in leadership roles to manifest or, alternatively, small sample size.

Importantly, when compared to national data on women faculty in academic medicine, we found that NIH study sections included higher proportions of professors (35% vs 29%) and similar proportions of associate professors (45% vs 43%) and assistant professors (48% vs 49%) as reviewers [20]. This finding suggests that women who are more established in their careers are more likely to be selected as reviewers. It also indicates that women professors as a group may take on a disproportionate share of the peer review workload. Such concentration of responsibilities on this smaller cohort may limit their bandwidth for other professional responsibilities or impact burnout in this group.

For limitations, this study examined one grant cycle (May–June) over three years, which may limit generalizability. Gender was binarily defined; if pronouns were unavailable, photo-based gender identification may have misgendered individuals. This study also lacked comprehensive data about reviewers. While we included gender, academic rank, reviewer role, and reviewer status, no data were available on characteristics such as race/ethnicity, years since completing the highest degree, or years of experience in the study section.

This study highlights the need for the NIH to provide comprehensive and publicly available data on study section reviewers’ characteristics, such as gender and race/ethnicity. Future research also should examine the potential effects of discrimination, biases, and policies in study sections, as well as potential drivers of positive change. Understanding and addressing the representation of scientists influencing NIH grant decisions is important to ensuring innovative science that meets the nation’s pluralistic needs.

## Supporting information

10.1017/cts.2025.10091.sm001 Alejandro et al. supplementary material Alejandro et al. supplementary material

## Supplementary material

The supplementary material for this article can be found at https://doi.org/10.1017/cts.2025.10091.
