# Supplementary material for: Representation of women on National Institutes of Health study sections before and during COVID-19 pandemic
Source: J Clin Transl Sci. 2025 Jul 7;9(1):e152. doi: 10.1017/cts.2025.10091 (PMC12392349; doi:10.1017/cts.2025.10091)
Supplement: Alejandro et al. supplementary material [file S2059866125100915sup001.docx]

**SUPPLEMENTARY MATERIAL**

Table. Likelihood of reviewers on National Institutes of Health study section being women, 2019-2021 May-June review cycles, excluding individuals whose gender was imputed

| **Subgroup and time period^a^** | **Risk ratio** | **95% Confidence interval** | **p-value** |
| --- | --- | --- | --- |
| **All reviewers (n=26,002)** |  |  |  |
| 2019 | 1 [ref] | NC | NC |
| 2020 | 1.003 | 0.97, 1.04 | 0.9 |
| 2021 | 1.06 | 1.03, 1.10 | 0.005 |
| **Permanent (n=17,433)** |  |  |  |
| 2019 | 1 [ref] | NC | NC |
| 2020 | 1.01 | 0.97, 1.05 | 0.7 |
| 2021 | 1.06 | 1.02, 1.10 | 0.007 |
| **Temporary (n=8,569)** |  |  |  |
| 2019 | 1 [ref] | NC | NC |
| 2020 | 0.99 | 0.93, 1.05 | 0.7 |
| 2021 | 1.07 | 1.01, 1.14 | 0.02 |
| **Chairperson (n=1170)** |  |  |  |
| 2019 | 1 [ref] | NC | NC |
| 2020 | 0.95 | 0.80, 1.13 | 0.6 |
| 2021 | 1.18 | 0.98, 1.41 | 0.08 |
| **Non-chairperson (n=24,832)** |  |  |  |
| 2019 | 1 [ref] | NC | NC |
| 2020 | 1.005 | 0.97, 1.04 | 0.8 |
| 2021 | 1.06 | 1.02, 1.09 | 0.001 |

^a^Poisson regressions assessed the likelihood that any given reviewer is a woman, adjusting for academic rank, status, and role. Random effects included study section and institute/center/office. Fixed effects included year, academic rank, status, and role. Changes over time were also stratified by status (permanent/temporary) or role (chairperson/non-chairperson) where noted. Total n for all reviewers and by stratified subgroups reflects all non-unique reviewers.

Abbreviations: Ref, reference group; NC, not calculated
